# Microglial reactivity predicts hippocampal, but not global, atrophy in cerebral small vessel disease

**DOI:** 10.1002/alz.71336

**Published:** 2026-04-02

**Authors:** Adriana Zainurin, Robin B. Brown, Daniel J. Tozer, Hugh S. Markus

**Affiliations:** ^1^ Stroke Research Group, Department of Clinical Neurosciences University of Cambridge Cambridge UK

**Keywords:** blood‐brain barrier, dementia, MRI, neuroinflammation, positron emission tomography, stroke, vascular cognitive impairment

## Abstract

**INTRODUCTION:**

Cerebral small vessel disease (CSVD) is the most prevalent pathology underlying vascular dementia. Increased neuroinflammation and blood‐brain barrier (BBB) permeability have been implicated in CSVD pathogenesis. We determined whether microglial reactivity and BBB permeability at baseline predicted whole‐brain and hippocampal atrophy over one year, and cognitive impairment over four years.

**METHODS:**

Seventy‐seven patients with CSVD were recruited to this prospective study. Baseline microglial reactivity and BBB permeability were determined using ^11^C‐PK11195 positron emission tomography and dynamic contrast enhanced (DCE) MRI respectively.

**RESULTS:**

Greater ^11^C‐PK11195 binding at baseline was associated with hippocampal atrophy over one year (*p *= 0.001), but not with global brain atrophy. Cox regression analyses showed no significant associations between ^11^C‐PK11195 and cognitive impairment. There were no associations between BBB permeability with whole‐brain and hippocampal atrophy, or with cognitive impairment.

**DISCUSSION:**

Our data suggests that microglial reactivity may play a role in hippocampal atrophy; potentially contributing to the increasingly recognized interaction between vascular and neurodegenerative pathology.

## Highlights

- Microglial activation may play a role in hippocampal atrophy in small vessel disease.
- We found no evidence that microglial activation predicts future cognitive impairment risk.
- BBB permeability was not associated with future risk of atrophy or cognitive impairment.

## 1 BACKGROUND

Cerebral small vessel disease (CSVD) causes a quarter of all strokes and is the most common pathology underlying vascular dementia.^1^ Most cases are sporadic, particularly related to hypertension, but a number are familial, the most common cause of which is Cerebral Autosomal Dominant Arteriopathy with Subcortical Infarcts and Leukoencephalopathy (CADASIL). Characteristic appearances of both sporadic and familial CSVD on magnetic resonance imaging (MRI) include white matter hyper intensities (WMH), lacunes, cerebral micro bleeds and brain atrophy. Despite its importance, there are no proven treatments for CSVD beyond cardiovascular risk factor control. A major reason for this is incomplete understanding about the underlying pathophysiology. More recently two processes, which could be potentially targeted therapeutically, have been implicated in CSVD pathogenesis: increased blood‐brain barrier (BBB) permeability and neuroinflammation.

Neuropathological and cerebrospinal fluid (CSF) studies have demonstrated leakage of plasma proteins into the brain consistent with BBB leakage in CSVD.^2^, ^3^ Using dynamic contrast‐enhanced MRI (DCE‐MRI), multiple studies have demonstrated changes consistent with BBB leakage in patients with various manifestations of CSVD including WMH, lacunar stroke and vascular cognitive impairment.^4^, ^5^, ^6^ Increased BBB leakage was not only found in areas of white matter lesions, but also in normal‐appearing white matter,^5^ while regional analysis has detected “hotspots” of increased BBB permeability.^7^ It has been hypothesized that BBB leakage results in extravasation of circulating neuroinflammatory molecules into the brain causing brain injury.^8^

Both systemic inflammation^9^ and CNS inflammation^7^, ^10^ (“neuroinflammation”) have also been implicated in CSVD. The former being inflammation occurring throughout the body, with the latter being restricted to inflammation in the brain and spinal cord. Microglial signal can be imaged in‐vivo in humans using positron emission tomography (PET) imaging with ^11^C‐PK11195, a radioligand with high‐affinity to the 18‐kDa translocator protein (TSPO).^11^, ^12^ Although TSPO is expressed in other cell types, measuring the binding potential of ^11^C‐PK11195 to TSPO as a proxy of microglial signal is widely used. Recent studies have demonstrated increased ^11^C‐PK11195 binding in CSVD patients with both lacunar stroke^7^, ^13^ and WMH.^14^

**RESEARCH IN CONTEXT**

1. **Systematic review**: There is emerging evidence that neuroinflammation and blood‐brain barrier (BBB) permeability have been implicated in cerebral small vessel disease (CSVD) pathogenesis. However, it remains unclear whether these pathogenic mechanisms play a causal role in CSVD, or whether they occur secondary to tissue damage.
2. **Interpretation**: Our results suggest that increased microglial activation (neuroinflammation) may play a role in hippocampal atrophy, but not global atrophy or risk of cognitive impairment. We found no evidence that increased BBB permeability predicts brain atrophy or cognitive impairment in CSVD.
3. **Future directions**: These findings underscore the potential value of microglial activation as a biomarker for predicting hippocampal atrophy—and possibly other imaging‐based risk factors–in CSVD. Furthermore, examining microglial activation alongside blood‐brain barrier permeability may help elucidate the temporal dynamics and mechanistic interplay between these pathological processes. Further research should investigate these mechanisms in a larger sample size and over a longer follow‐up duration.

While both increased BBB permeability and increased ^11^C‐PK11195 binding have been demonstrated in CSVD, the key question is whether either of these processes is causal, or whether they occur secondary to tissue damage. Only if they are causal is it likely that targeting these processes will delay disease progression. Data from an animal model of white matter ischemia suggests that the two processes are inter‐related and part of a cascade that can be inhibited pharmacologically, resulting in reduced white matter damage and improved neurological function.^10^ However proving causality in humans requires longitudinal studies demonstrating that these changes predict disease progression, prior to therapeutic intervention studies. There are few such studies to date. One study in 43 patients showed an association between BBB leakage at baseline and the loss of microstructural integrity over two years in the perilesional zones around WMHs.^15^ While few studies have examined whether ^11^C‐PK11195 predicts cognitive decline in CSVD, microglial signal was associated with brain atrophy^16^ and predicted cognitive decline^17^ in Alzheimer's disease (AD).

We hypothesized that brain inflammation predicts both clinical and radiological disease progression in CSVD. To address this question, we determined whether ^11^C‐PK11195 binding and BBB permeability at baseline predicted cognitive impairment over a four‐year follow‐up period, in patients with both sporadic and monogenic (CADASIL) CSVD. We also determined whether they predicted brain atrophy over a one‐year follow‐up on repeat MRI. Brain atrophy has been shown to be a sensitive marker to detect change in CSVD.^18^ In addition, we also examined associations with hippocampal atrophy in a prespecified analysis for the following reasons. Firstly, hippocampal atrophy is a feature in both sporadic and genetic (CADASIL) CSVD, and the degree of hippocampal atrophy correlates with the degree of cognitive impairment.^19^, ^20^ Secondly, studies have shown that the hippocampus is particularly sensitive to the effects of inflammation.^21^, ^22^ Thirdly, increasing evidence suggests that there may be an interaction between CSVD and other neurodegenerative pathologies.^23^ While this may be simply an additive effect, it has been suggested that there may be an interaction between the two pathologies, and we hypothesized this might be due to CSVD‐related inflammation increasing hippocampal damage. Hippocampal damage is a hallmark of AD.

## 2 METHODS

### 2.1 Patients

The study involved prospective follow‐up of 77 patients with symptomatic CSVD (sporadic CSVD, *n*  =  57; CADASIL, *n*  =  20) who all underwent an identical imaging and clinical protocol. Forty of the patients were involved in an observational phase of the study^7^ whereas the remaining 37 patients were involved in a randomized control trial (“MINERVA”).^13^ Nineteen patients in the MINERVA study received minocycline while the remaining participants received a placebo. The results of the MINERVA trial found no treatment effect on ^11^C‐PK11195 binding and BBB permeability^13^; hence, these participants were included in the present study. However, the analysis was repeated without these subjects to assess whether the remaining participants behaved in the same way.

For patients with sporadic CSVD, the inclusion criteria were clinical evidence of a lacunar stroke syndrome, with a corresponding lacunar infarct either on diffusion‐weighted imaging for cases imaged within three weeks of stroke, or for cases imaged later after stroke an anatomically compatible lacunar infarct (≤ 1.5 cm in diameter) on FLAIR/T1 MRI. In addition confluent WMH, defined as a rating of ≥ 2 on the Fazekas scale, was required.^24^ Patients with CADASIL had a confirmed genetic diagnosis of CADASIL with a typical cysteine‐changing *NOTCH3* mutation. The patient cohorts were recruited from a comprehensive in‐ and outpatient stroke service, and a national CADASIL clinic, both based at Addenbrooke's Hospital, Cambridge, UK.

Exclusion criteria included severe cognitive impairment (defined as a MMSE < 21) to ensure that patients could comply with the protocol, contraindication to MRI, females who were of childbearing age and/or who were breastfeeding and an estimated glomerular filtration rate (eGFR) ≤59 mL/min/1.73 m^2^ due to concerns regarding gadolinium administration in the presence of renal impairment. All participants were studied at least three months after most recent clinical stroke to avoid changes in inflammation or BBB permeability related to acute ischemia.

Written informed consent was obtained from all patients. The observational phase of the study was approved by the East of England—Cambridge South Ethics Committee (REC no: 16/EE/0468, IRAS project ID: 212632), and the Administration of Radioactive Substances Advisory Committee (ARSAC ref: 83/3886/35752). The MINERVA trial was approved by the Cambridge Central Research Ethics Committee (reference 18/EE/0237) and the UK Administration of Radioactive Substances Advisory Committee (ARSAC, Research ID 176; September 19, 2018). MINERVA was registered on the International Clinical Trials Registry Portal (reference ISRCTN15483452).

### 2.2 Neuroimaging acquisition

Neuroimaging PET and MRI data were acquired in a single session on the GE SIGNA PET/MR scanner (GE Healthcare, Waukesha, USA) at the Wolfson Brain Imaging Centre in Cambridge, UK. The scanner can simultaneously acquire PET and 3T MRI data. Scans were acquired using a 32‐channel NOVA head coil (NOVA medical).

Non‐contrast MRI sequences included: (i) 3D T1‐weighted images taken with a fast spoiled gradient echo sequence; flip angle = 12°, inversion time = 450 ms, field of view = 28 mm, slice thickness = 1 mm, number of slices = 192, reconstructed matrix size = 512 × 512, (ii) axial susceptibility weighted imaging; flip angle = 17°, repetition time = 40.6 ms, echo time = 24.2 ms, field of view = 22 mm, slice thickness = 2 mm, number of slices = 70, reconstructed matrix size = 256 × 256, (iii) axial T2 FLAIR sequence; flip angle = 160°, repetition time = 8800 ms, echo time = 120 ms, inversion time = 2445 ms, field of view = 22 mm, slice thickness = 5 mm, number of slices = 28, reconstructed matrix size = 256 × 256, (iv) axial diffusion tensor imaging (DTI); echo time = minimum, repetition time = 15763 ms, field of view = 19.2 mm, slice thickness = 2 mm, number of slices = 665–670, reconstructed matrix size = 256 × 256, (v) axial T2 fast spoiled gradient echo sequence; flip angle = 111°, echo time = 85 ms, repetition time = 6000 ms, field of view = 22 mm, slice thickness = 5 mm, number of slices = 31, reconstructed matrix size = 1024 × 1024.

Dynamic contrast‐enhanced MRI (DCE‐MRI) was acquired following the non‐contrast MRI. Gadolinium was injected intravenously at a dose of 0.025 mmol/kg at an injection rate of 6 mL/s. T1 was mapped prior to injection, followed by 22 min of T1 mapping post‐injection. To obtain T1 relaxation times, the T1 mapping sequence uses a 3D radiofrequency spoiled gradient echo imaging sequence. Six flip angles were used to calculate each map: 2°, 5°, 12°, 17°, 22°, 27°. Eight post‐injection maps with a temporal resolution of ∼2.5 min were collected. Repetition time = 6.3 ms, echo time = 1.784 ms, resolution = 2 × 2 × 3 mm (reconstructed to 0.94 × 0.94 × 3 mm), number of slices = 16, reconstructed matrix size = 256 × 256. Prior to injection, a B0 mapping sequence was acquired for flip angle correction; flip angle = 15°, number of echoes = 1, receiver bandwidth = 15.63, field of view = 35 mm, slice thickness = 5 mm.

PET imaging was co‐acquired after injection of the radioligand ^11^C‐PK11195, produced at the Wolfson Brain Imaging Centre Radiopharmaceutical Unit. The radioligand was injected over 30s and list‐mode PET data were acquired for 75 min. The median injected activity was 440 MBq (interquartile range [IQR] 40–483 MBq) with corresponding injected mass values of 3.9 (IQR 2.8–6.4) µg.

### 2.3 Non‐contrast MRI analysis

WMH volume was quantified on FLAIR images by a single trained rater using the semi‐automatic contouring technique Jim analysis software version 7.0.5 (Xinapse Systems Limited). Whole brain WMH maps were then generated. A second experienced rater remarked ten FLAIR scans in a randomized, blinded setting. The inter‐rater and intra‐rater reliability coefficients were 0.988 and 0.993, respectively. To calculate WMH lesion load, WMH volume was normalized to total brain volume and expressed as a percentage. Lacunes were identified on FLAIR images by a single neurologist, blinded to subject identity. Both T1 and FLAIR scans were visually inspected to confirm the presence of lacunes. The FLAIR image was registered to the T1 image using a rigid body transformation in Advanced Normalization Tools (ANTs; http://stnava.github.io/ANTs/).

Brain volumes were calculated from T1 images. T1 images were processed using the “segment” routine in SPM12 (https://www.fil.ion.ucl.ac.uk/spm/software/spm12/). SPM segmentation provides tissue probability maps. Volumes for each tissue class were calculated as the sum of all voxels that have a probability of > 0.5 of belonging to that class, after removal of voxels in the WMH mask. The tissue segments and WMH mask were used to create the NAWM and WM masks whereby WM masks are the sum of NAWM and WMH masks. The masks were then eroded by 3 mm using *fslmaths* (https://fsl.fmrib.ox.ac.uk/fsl/fslwiki), to eliminate ventricular or grey matter contamination.

Percentage whole brain volume change in each patient was calculated using *SIENA* ^25^, ^26^ using T1‐weighted MR images obtained at baseline and one‐year follow‐up. Percentage hippocampal volume change was obtained by first performing brain segmentation using *Freesurfer* (version 7.4.0), then computed by dividing the change in hippocampal volume by total brain volume.

### 2.4 Contrast MRI analysis

Permeability maps were created using previously published methods.^27^ T1 maps from contrast (dynamic contrast enhanced; DCE) MRI are used to calculate estimates of gadolinium concentration in tissue, assuming a linear relationship. The influx rate (*K*_i_) as a metric of permeability was determined using Patlak graphical analysis which was applied to the gadolinium concentration images. Global values of BBB permeability in the WM, NAWM and WMH masks were calculated as the mean *K*_i_ values across voxels in that region. “Hotspots” were classified as voxels with a *K*_i_ value greater than the 95^th^ percentile of the *K*_i_ distribution in NAWM of a control population scanned using the same sequence and scanner. Hotspot maps were generated using tissue segments and *K*_i_ maps with the threshold. The volumes of hotspots were then determined.

### 2.5 PET analysis

List‐mode PET data were histogram med into 55 time‐frames and then reconstructed into images (128 × 128 × 89 matrix; 2.0 × 2.0 × 2.8 mm voxel size) using time‐of flight ordered subsets expectation maximization with 16 subsets, 6 iterations and no smoothing. Attenuation correction was applied using a multi‐subject atlas method and improvements to the brain coil component. Image reconstruction included correction for random coincidences, dead time, normalization, scattered coincidences, radioactive decay, and sensitivity.

For global ^11^C‐PK11195 binding, SPM12 was used to realign each dynamic image series and reduce the impact of head motion. A mean realigned PET image was then used to co‐register each realigned dynamic PET image series to the T1 BRAVO MR image from the same scan. To estimate specific ^11^C‐PK11195 binding, binding potential relative to a non‐displaceable compartment (BP_ND_) was determined with a basis function version of the simplified reference tissue model incorporating correction for vascular binding, using a white matter segment as estimated with supervised cluster analysis using library data determined from control scans of another ^11^C‐PK11195 project on the same scanner with the same acquisition and processing protocol. Global mean and hotspot maps of BP_ND_ were determined in the same way as BBB permeability; for the hotspot threshold, the 95^th^ percentile of the BP_ND_ distribution in the control population was used.^7^ The derived metrics of microglial signal and BBB permeability were further classified into type of measure (global mean and hotspot volume) and brain tissue of interest (NAWM and all WM).

### 2.6 Follow‐up data collection

Patients were prospectively followed up with neuroimaging, cognitive and clinical evaluations. Non‐contrast MRI acquisition was repeated one year after baseline scan on the same PET/MR scanner. Cognitive performance was assessed using the Montreal Cognitive Assessment (MoCA)^28^ at four years post‐baseline visit. Progression to mild cognitive impairment or dementia up to four years follow‐up was recorded by at clinical assessments and review of electronic medical records.

### 2.7 Definition of cognitive endpoints

Dementia was diagnosed using the DSM‐5^29^ definition of major neurocognitive disorder and was present if individuals met at least one of the following criteria: (i) diagnosis of dementia made in a memory clinic or equivalent clinical service, (ii) adjudication by a neurologist who agreed that the clinical picture met the DSM‐5 criteria or MoCA scores ≤ 18). Patient responses from Disability Assessment for Dementia (DAD) informant‐rated questionnaires were collected to evaluate the patient's basic and instrumental activities in daily activities and functional disability.^30^ During the adjudication process, medical records, DAD scores of each patient were reviewed by a neurologist who was blinded to MRI and risk information.

Mild cognitive impairment was defined as a MoCA score of ≤ 23.^31^, ^32^ In this study, “cognitive impairment” was defined as patients who either had mild cognitive impairment (based on MoCA scores as above) and/or a dementia diagnosis.

### 2.8 Statistical analyses

Descriptive data are presented as mean (SD) for continuous variables and as frequency (%) for categorical variables. Continuous variables were compared between the sporadic CSVD and CADASIL groups using independent t‐tests whereas categorical variables were compared using chi‐square (χ^2^) tests.

Linear regression models, adjusted for age and sex, were employed to examine the associations of neuroinflammatory markers, BBB leakage markers, and conventional MRI markers with percentage volume changes in the whole brain and hippocampus. Normality of variables was assessed using the Shapiro‐Wilk test, and the percentage hippocampal volume change was cube‐root transformed to achieve a normal distribution. To standardize units and facilitate comparison, all variables were scaled. Analyses were conducted for the entire cohort with group (sporadic CSVD and CADASIL) as a covariate. If a significant association was found, then the analysis was additionally controlled for other confounders such as WMH lesion load, number of cerebral micro bleeds, number of lacunes and number of years in education. We then ran the analysis for each group separately.

To investigate the associations of neuroinflammatory markers, BBB leakage markers, and conventional MR markers with incident cognitive impairment, Cox proportional hazard regression models adjusted for age, sex and group (sporadic CSVD or CADASIL) were performed. Again, the analysis was repeated for each group (without group as a covariate). Time to cognitive impairment was defined as the interval between the baseline visit and the date of clinical diagnosis or the MoCA assessment. Analyses were conducted on all patients with follow‐up data, whether from cognitive assessment (MoCA), clinical dementia diagnosis, or both. All statistical analyses were performed using R statistics (version 4.3.2 2023, R Foundation for Statistical Computing).

## 3 RESULTS

### 3.1 Cohort details

**FIGURE 1 alz71336-fig-0001:**
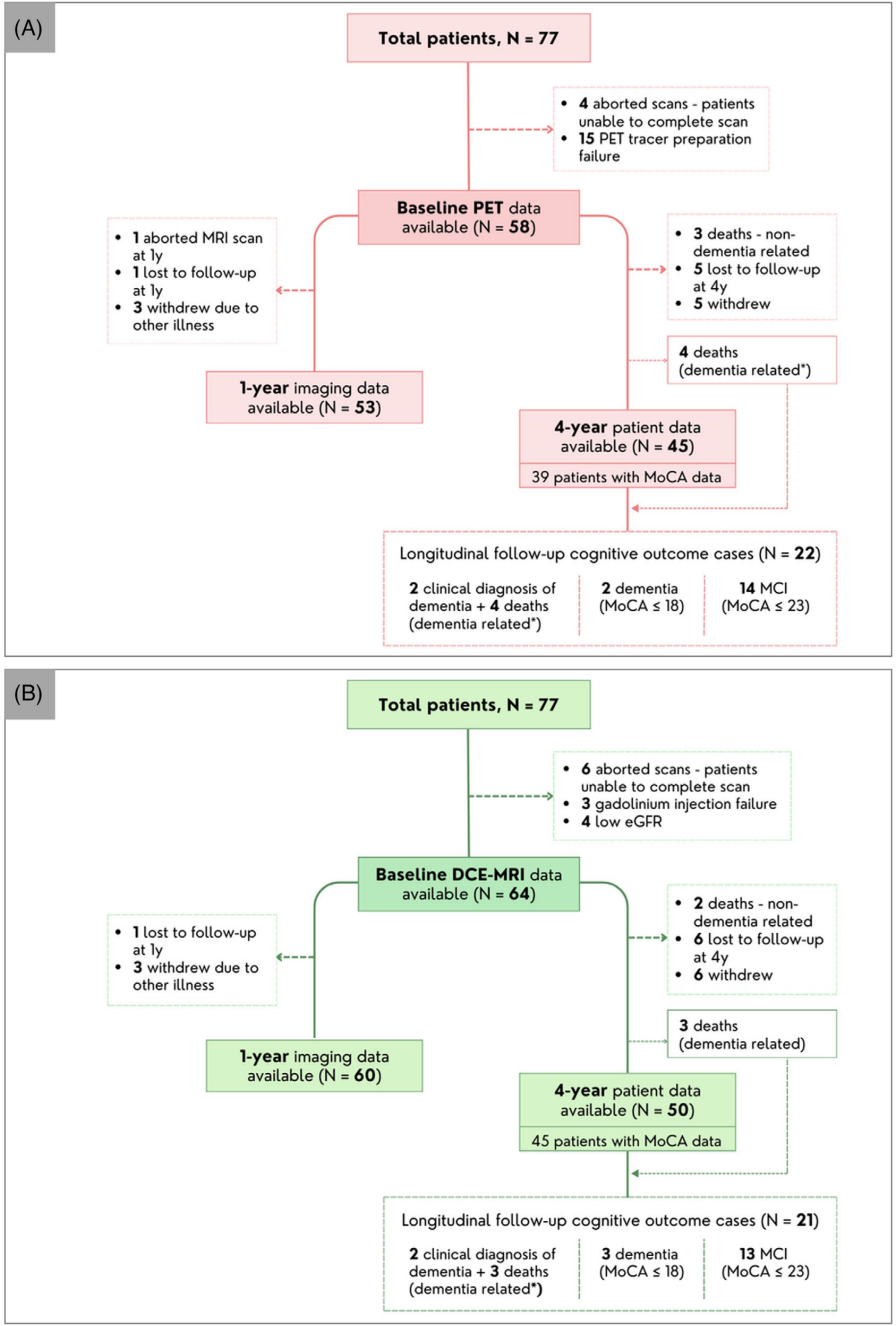
Flowchart of (A) PET and (B) DCE‐MRI data availability at baseline, 1‐year (1y) and 4‐year (4y) longitudinal follow‐up. DCE‐MRI, dynamic contrast enhanced MRI; eGFR, estimated glomerular filtration rate; MCI, mild cognitive impairment; PET, positron emission tomography.

Demographic, clinical, and MRI characteristics of the patients and demographic and clinical characteristics of the controls used for the hot‐spot determination are summarized in Table 1. Mean age was 65.3 years, and 40% were female. Baseline PET and DCE‐MRI data was available for 58 and 64 patients of the 77 patients respectively. Figure 1 depicts a flowchart detailing the excluded data from the overall cohort.

**TABLE 1:** Patient characteristics at baseline.

	All (*n* = 77)	Sporadic CSVD (*n* = 57)	CADASIL (*n* = 20)	Controls (*n* = 20)
**Demographics**				
Age, years	65.3 (12.9)	70.00 (10.54)	51.85 (9.46)	66.4 (6.7)
Sex (female)	31 (40%)	24 (42%)	7 (35%)	7 (35%)
Education, years	13.74 (2.97)	13.61 (3.27)	14.10 (3.28)	13.4 (3.8)
Ethnicity, (White/Caucasian)	68 (88%)	52 (68%)	16 (80%)	200 (100%)
Time from stroke to scan, months	21.21 (20.94)	18.71 (19.84)	38.71 (21.54)	–
**Risk factors**				
Hypertension	58 (75%)	50 (88%)	8 (40%)	6 (30%)
Systolic blood pressure, mmHg	139.27 (22.94)	146.61 (22.60)	126.05 (17.22)	142.05 (17.70)
Diastolic blood pressure, mmHg	75.63 (12.40)	76.31 (13.11)	74.40 (11.22)	81.21 (9.03)
Hyperlipidaemia	45 (58%)	42 (74%)	3 (15%)	7 (35%)
Ischaemic heart disease	4 (5.2%)	4 (7.0%)	0 (0%)	0 (0%)
Smoking history	40 (52%)	33 (58%)	7 (35%)	12 (60%)
Depression	25 (32%)	17 (30%)	8 (40%)	7 (35%)
Migraine	33 (43%)	17 (30%)	16 (80%)	5 (25%)
Diabetes (Type 2)	11 (14%)	11 (19%)	0 (0%)	1 (5%0
**Imaging markers**				
Intracranial volume, cm^3^	1580.70 (175.87)	1578.68 (185.74)	1586.66 (147.60)	
Total brain volume, cm^3^	1290.15 (198.36)	1308.22 (206.98)	1241.36 (168.03)	
Hippocampal volume, cm^3^	8.44 (1.31)	8.10 (1.20)	9.43 (1.15)	
Gray matter volume, cm^3^	664.22 (118.68)	653.15 (102.12)	694.12 (154.07)	
All white matter volume, cm^3^	99.27 (36.92)	90.83 (34.31)	119.96 (35.62)	
Normal appearing white matter volume, cm^3^	65.54 (24.71)	64.63 (24.89)	67.76 (24.77)	
WMH volume, cm^3^	36.92 (31.69)	27.97 (22.40)	61.08 (40.22)	
WMH lesion load (%)	2.90 (2.50)	2.15 (1.64)	4.99 (3.29)	
Cerebral microbleeds; n (range)	0 (0–82)	0.5 (0–82)	0 (0–60)	
Lacunes; n (range)	2 (0–26)	2 (0–8)	2 (0–26)	
^11^C‐PK11195 binding global mean in NAWM	−0.04 (0.04)	−0.04 (0.03)	−0.05 (0.06)	
^11^C‐PK11195 binding global mean in all WM	−0.07 (0.04)	−0.07 (0.03)	−0.08 (0.05)	
^11^C‐PK11195 binding hotspot volume in NAWM, cm^3^	14.9 (23.3)	13.3 (23.7)	19.9 (22.3)	
^11^C‐PK11195 binding hotspot volume in all WM, cm^3^	7.5 (8.4)	7.3 (8.0)	8.0 (10.0)	
BBB K_i_ global mean in NAWM (× 10^−4^ mL/g/min) BBB K_i_ global mean in all WM (× 10^−4^ mL/g/min)	4.0 (7.6)	5.0 (8.7)	2.0 (2.9)	
	3.8 (7.7)	5.0 (8.9)	2.0 (2.7)	
BBB K_i_ hotspot volume in NAWM, cm^3^	5.35 (6.57)	5.92 (6.58)	3.99 (6.54)	
BBB K_i_ hotspot volume in all WM, cm^3^	7.01 (7.81)	7.63 (8.02)	5.56 (7.29)	

Values represent mean (S.D.) for continuous variables and frequency (percentage) for categorical variables.

Abbreviations: K_i_, influx rate; NAWM, normal appearing white matter; WM, white matter; WMH, white matter hyperintensities.

For the analysis of changes in brain volume at the one‐year follow‐up timepoint, there were 53 (PET data) and 60 (DCE‐MRI data) patients with both baseline and follow‐up data (Figure 1). The mean duration of between baseline and 1‐year follow‐up imaging was 12.33 months (Table 1). For the longitudinal follow‐up regarding progression to cognitive impairment, data was available for 45 and 50 patients with PET and DCE‐MRI respectively (Figure 1). Statistical analyses showed no significant difference in demographics between groups of patients included and excluded in the analyses (Tables S1–S2). However, there were less lacunes and cerebral micro bleeds in the excluded cases (see Tables S1–S2).

### 3.2 Whole brain and hippocampal atrophy

Mean time to repeat MRI was 12.33 (SD 0.96) months. Over this period, mean percentage brain volume change was −0.49% (0.93), whereas mean hippocampal volume change was −1.02% (1.64). The results of linear regressions examining associations between ^11^C‐PK11195 binding and DCE‐MRI markers with percentage whole brain volume change and hippocampal volume are shown in Tables 2 and 3 respectively. Associations with conventional MRI markers are shown in Supplementary Table S3 and S4 respectively.

**TABLE 2 alz71336-tbl-0002:** Associations between ^11^C‐PK11195 binding and DCE‐MRI markers with percentage whole brain volume change.

	Brain tissue	Whole group			Sporadic CSVD (PET, *n* = 40; DCE‐MRI, *n* = 43)			CADASIL (PET, *n* = 13; DCE‐MRI, *n* = 17)		
Predictor		*β*	95% CI	*p* value	*β*	95% CI	*p* value	*β*	95% CI	*p* value
**Neuroinflammatory markers (PET)**										
Global mean	NAWM	−0.19	−0.47, 0.08	0.156	−0.1	−0.42, 0.21	0.513	−0.41	−1.03, 0.21	0.166
	All WM	−0.09	−0.33, 0.19	0.51	−0.17	−0.48, 0.15	0.289	0.51	−0.48, 1.5	0.275
Hotspot volume	NAWM	−0.06	−0.34, 0.22	0.671	0.05	−0.27, 0.37	0.756	−0.37	−1.03, 0.29	0.239
	All WM	−0.15	−0.42, 0.13	0.292	−0.03	−0.36, 0.29	0.834	−0.4	−1.06, 0.25	0.195
**Blood‐brain barrier leakage markers (DCE‐MRI)**										
Global mean	NAWM	0.09	−0.17, 0.35	0.477	0.06	−0.25, 0.36	0.711	0.16	−0.42, 0.73	0.571
	All WM	0.1	−0.16, 0.35	0.461	0.05	−0.25, 0.36	0.72	0.19	−0.38, 0.77	0.481
Hotspot volume	NAWM	0.02	−0.24, 0.28	0.852	0.04	−0.27, 0.35	0.786	−0.13	−0.75, 0.48	0.644
	All WM	0.02	−0.24, 0.27	0.907	0.03	−0.27, 0.34	0.822	−0.13	−0.73, 0.47	0.647

Table shows standardised *β* coefficients, 95% confidence intervals (CI) and *p* values of linear regression models. Models have been adjusted for age and sex.

Abbreviations: NAWM, normal appearing white matter; WM, white matter.

**TABLE 3 alz71336-tbl-0003:** Associations between ^11^C‐PK11195 binding and DCE‐MRI markers with percentage hippocampal volume change.

	Brain tissue	Whole group			Sporadic CSVD (PET, *n* = 40; DCE‐MRI, *n* = 43)			CADASIL (PET, *n* = 13; DCE‐MRI, *n* = 17)		
Predictor		*β*	95% CI	*p* value	*β*	95% CI	*p* value	*β*	95% CI	*p* value
**Neuroinflammatory markers (PET)**										
Global mean	NAWM	−0.14	−0.43, 0.14	0.311	−0.09	−0.41, 0.24	0.579	−0.28	−0.97, 0.41	0.381
	All WM	0.1	−0.19, 0.39	0.487	0.02	−0.31, 0.35	0.902	−0.07	−1.18, 1.05	0.898
Hotspot volume	NAWM	−0.43	−0.69, −0.17	0.002	−0.62	−0.87, −0.37	1.3×10−^5^	−0.02	−0.77, 0.74	0.957
	All WM	−0.15	−0.44, 0.13	0.283	−0.14	−0.47, 0.19	0.406	−0.09	−0.84, 0.67	0.798
**Blood‐brain barrier leakage markers (DCE‐MRI)**										
Global mean	NAWM	−0.05	−0.33, 0.23	0.735	−0.13	−0.45, 0.18	0.398	0.23	−0.35, 0.81	0.413
	All WM	−0.03	−0.31, 0.25	0.833	−0.13	−0.45, 0.19	0.433	0.26	−0.32, 0.84	0.346
Hotspot volume	NAWM	−0.17	−0.44, 0.11	0.231	−0.15	−0.47, 0.18	0.367	−0.1	−0.73, 0.53	0.735
	All WM	−0.15	−0.4,2 0.13	0.295	−0.11	−0.43, 0.21	0.476	−0.1	−0.72, 0.52	0.724

Table shows standardised *β* coefficients, 95% confidence intervals (CI) and *p* values of linear regression models. Models have been adjusted for age and sex.

Abbreviations: NAWM, normal appearing white matter; WM, white matter.

^*^ *p *< 0.05.

^**^ *p *< 0.01.

^***^ *p *< 0.001.

There were no associations between ^11^C‐PK11195 binding and percentage whole brain volume change. In contrast, a significant association was found between NAWM ^11^C‐PK11195 binding hotspot volume and percentage hippocampal volume change in the whole group (*β* = −0.43, 95% CI = −0.69, −0.17, *p *= 0.002; Table 3). The association remained significant (*β* = −0.34, 95% CI = −0.65 to −0.02, *p* = 0.035) after further adjusting for WMH lesion load, number of cerebral microbleeds and number of lacunes and survived false discovery rate (FDR) correction (FDR‐corrected *p* = 0.008). On division into CSVD subtypes, the association was present in the sporadic CSVD group (*β* = −0.62, 95% CI = −0.87 to −0.37, *p* = 1.3 × 10^−5^; Table 3); this association remained significant after controlling for the same covariates (*β* = −0.42, 95% CI = −0.76 to −0.07, *p* = 0.019) and survived FDR correction (FDR‐corrected *p* = 0.000104). In contrast there was no association in the CADASIL group (*β* = −0.02, 95% CI −0.77 to 0.74, *p* = 0.957; Table 3). Secondary analyses showed no significant associations between baseline ^11^C‐PK11195 binding with percentage change in WMH volume (Table S5) or percentage change in diffusion tensor imaging markers–mean diffusivity and fractional anisotropy–over one year (Tables S6 and S7).

Supplementary Tables S8–S10 replicate Tables 2, 3, 4 but without the participants who received minocycline. As can be seen the pattern of results is retained with a significant relationship between NAWM ^11^C‐PK11195 binding hotspot volume and percentage hippocampal volume change.

**TABLE 4 alz71336-tbl-0004:** Associations between ^11^C‐PK11195 binding and DCE‐MRI markers with cognitive impairment.

		Whole group			Sporadic CSVD			CADASIL		
Predictor	Brain tissue	Incident cases, n	Hazard ratio [95% CI]	*p* value	Incident cases, n	Hazard ratio [95% CI]	*p* value	Incident cases, *n*	Hazard ratio [95% CI]	*p* value
**Neuroinflammatory markers (PET)**										
Global mean	NAWM	22/45	1.22 [0.81, 1.84]	0.335	17/32	1.01 [0.65, 1.57]	0.974	5/13	2.21 [0.7, 6.96]	0.175
	All WM	22/45	1.08 [0.69, 1.69]	0.748	17/32	1.01 [0.6, 1.69]	0.976	5/13	1.19 [0.38, 3.72]	0.76
Hotspot volume	NAWM	22/45	1.05 [0.78, 1.41]	0.754	17/32	0.9 [0.65, 1.25]	0.528	5/13	3.78 [1.21, 11.8]	0.022
	All WM	22/45	1.06 [0.79, 1.44]	0.692	17/32	0.88 [0.63, 1.23]	0.442	5/13	4.07 [1.22, 13.6]	0.023
**Blood‐brain barrier leakage markers (DCE‐MRI)**										
Global mean	NAWM	21/50	0.82 [0.37, 1.81]	0.622	17/32	1.01 [0.42, 2.46]	0.979	5/13	0 [0, 7.76]	0.152
	All WM	21/50	0.86 [0.35, 2.12]	0.741	17/32	1.1 [0.4, 3.03]	0.851	5/13	0 [0, 7.57]	0.146
Hotspot volume	NAWM	21/50	0.83 [0.5, 1.37]	0.462	17/32	0.99 [0.64, 1.52]	0.948	5/13	0.01 [0, 4.25]	0.137
	All WM	21/50	0.79 [0.49, 1.29]	0.353	17/32	0.99 [0.63, 1.55]	0.953	5/13	0.09 [0, 2.61]	0.163

Table shows standardised incident cases, hazard ratio, 95% confidence intervals (CI) and p‐values of Cox proportional hazard models. Models have been adjusted for age and sex. The total number of patients included in the analyses is detailed in Figure 1.

NAWM, normal appearing white matter; WM, white matter.

There were no significant associations between any DCE‐MRI markers with either percentage whole brain volume change or percentage hippocampal volume change. Additionally, in the secondary analyses, DCE‐MRI markers were not associated with percentage WMH volume change (Table S5) or percentage mean diffusivity change (Table S6). A significant association was found between baseline BBB mean and fractional anisotropy change over one year (*p* = 0.022; Table S7), but this association did not survive FDR correction (FDR‐corrected *p* = 0.088).

### 3.3 Longitudinal follow‐up of cognitive function

Mean time[Fig alz71336-fig-0001] from baseline to final clinical follow‐up was 53.73 (SD 20.06) months. Of the subjects with PET (*n* = 45) and DCE‐MRI (*n* = 50) data available for the longitudinal analyses, 22/45 (PET data) and 21/50 (DCE‐MRI data) patients were classified as having any “cognitive impairment” defined as either had mild cognitive impairment and/or a dementia diagnosis (Figure 1). Cox proportional hazards analyses showed no association between ^11^C‐PK11195 binding or DCE‐MRI markers with cognitive impairment in the whole population (Table 4). There were marginally significant associations in the CADASIL group, but these did not survive FDR correction and there were a small number of subjects and incident cases in this group. Associations with conventional MRI markers are shown in Supplementary Table S11.

## 4 DISCUSSION

In this longitudinal[Table alz71336-tbl-0002], [Table alz71336-tbl-0003], [Table alz71336-tbl-0004] study we found a significant association between ^11^C‐PK11195 binding, a marker of microglial signal, and hippocampal atrophy over one year, but not with global brain atrophy. Additionally, we found no significant association between ^11^C‐PK11195 binding and cognitive impairment over four years follow‐up. Previous cross‐sectional studies have shown evidence of neuroinflammation in CSVD^7^, ^13^, ^14^ but whether it is playing a casual role, and therefore represents a therapeutic target, or is merely secondary to tissue damage, remains controversial. Our data does not provide strong support for microglial reactivity playing a causal role in brain injury in CSVD, but raises the possibility that it may play a role in hippocampal atrophy, and perhaps the increasingly recognized interaction between vascular and neurodegenerative pathology.^33^

Hippocampal atrophy is an early neuropathlogical feature in AD but has also been described as a feature of both sporadic and genetic forms of CSVD, in both of which the degree of hippocampal atrophy has been found to correlate with the degree of cognitive impairment.^19^, ^20^ This has led to the suggestion that “hippocampal atrophy is an important pathway of cognitive impairment in vascular as well as degenerative disease”.^19^ The hippocampus is particularly susceptible to inflammation which could therefore play a role in CSVD‐related hippocampal damage, particularly at an early stage of the disease.^34^, ^35^ A higher susceptibility of the rodent hippocampal CA1 pyramidal neurons hippocampus to peripheral inflammatory stimuli, as compared to the cortex was found.^36^ Evidence from animal‐model studies have demonstrated that expression levels of microglia biomarkers was greater in the hippocampus compared to other brain regions following induced hypertension^34^ and stroke.^35^ Based on this evidence, it could be postulated that the hippocampus is more susceptible to inflammation‐related atrophy at an earlier stage of the disease progression compared to other brain regions in CSVD. Furthermore, a post‐mortem study involving community‐based participants showed that hippocampal microglial inflammation was significantly associated with late‐life cognitive decline and that it explained a large proportion (47%) of the direct effect in this relationship.^37^ The remaining indirect effect was accounted for by tau tangles and transactive response DNA‐binding protein 43 (TDP‐43) pathologies in separate pathways. This finding suggests that microglial inflammation in the hippocampus may contribute to cognitive decline via a distinct biological process independent of other neurodegenerative pathologies.^37^ Knowledge of the presence of tau (or other Alzheimer's pathologies) in these patients would have allowed us to determine the contributions of these to the atrophy and cognitive decline seen. Unfortunately, this was not acquired for this study which focused only on CSVD.

Considerable data has demonstrated that patients with AD pathology are more likely to develop clinical dementia in the presence of CSVD pathology.^23^ The relationship between neuroinflammation and hippocampal atrophy demonstrated in our study could provide a mechanism underlying this association. Studies with larger samples sizes and longer follow‐up are required to determine if increased ^11^C‐PK11195 binding is associated not only with hippocampal atrophy, but also with global atrophy and cognitive impairment over a longer follow‐up duration. It has also been shown in several papers^7^, ^13^ that specific cognitive domains are affected differently as a response to CSVD pathology, with executive function and processing speed particularly susceptible to decline. The current study does not have data on individual cognitive domains, but this could provide more specific information than a binary outcome of dementia conversion.

In contrast to inflammation, we found no association with any BBB permeability markers derived from DCE‐MRI, and either global or hippocampal atrophy over one year or risk of developing cognitive impairment over four years. This provides no support for increased BBB permeability playing a causal role in either brain atrophy or cognitive impairment in CSVD. While BBB permeability has been reported in several studies in CSVD, there is little data investigating whether it predicts disease progression. Findings from one small study suggested that BBB leakage at baseline was related to DTI changes over a two year period.^15^ This lack of sensitivity to predicting atrophy changes could represent a limitation of the technique to measure BBB permeability or physiological variation of the BBB permeability measurements over time. However, in the same dataset, we have previously shown that MRI measures of BBB permeability correlates well with the serum‐CSF albumin ratio, which estimates direct leakage from blood into the CSF,^13^ for a subset of 12 of the participants, partially validating the technique. Both the imaging analysis techniques would allow a regional, or voxel, based analysis approach and this would be an interesting approach for future work.

^11^C‐PK11195 binding has been used here as a marker of neuroinflammation, however it should be noted that this is an indirect pathway. A recent transcriptomic study has suggested that TSPO relates to microglial concentration rather than phenotype.^38^ Indeed, as other cells have TSPO binding sites it is a measure of protein density rather than microglial density or activation. Nevertheless, since the binding measure has been validated as a marker of inflammation,^39^ it is a viable measure to be used as indicative of neuroinflammation despite other contributions to the strength of binding.

The pathway from microglial reactivity to hippocampal atrophy is unclear. It is possible that the processes are largely unrelated, but both a product of underlying pathologies, however it is also possible that atrophy caused by cell death, is a result of the release of pro‐inflammatory cytokines and reactive oxygen species,^40^ or by triggering signaling pathways such as apoptosis.^41^

The sporadic CSVD and CADASIL populations behaved differently with regard to hippocampal atrophy. Apart from the difference in sample sizes, it is possible that this is due to the wider variation in response to ^11^C‐PK11195 seen in the CADASIL group.^7^ This suggests that there may be different disease pathways for sporadic CSVD and CADASIL, and that atrophy seen in both CSVD types possibly arises from different causal pathways. Lastly, the different age range of participants in the two groups may affect the extent of the underlying damage in the brain due to aging, consequently affecting the relationship.

The supplementary material (Tables S3 and S4) shows the conventional MRI markers of CSVD have limited relationships with the atrophy seen here with the only non‐volume related relationship being between lacune number and whole brain atrophy. Supplementary Table S11 shows more associations between the conventional markers and dementia development, although these do not survive FDR correction. These results suggest that the main measures discussed here are generally not better predictors than the conventional markers, although binding hotspot volume does show promise.

Our study has several strengths. We collected data on both BBB permeability markers and ^11^C‐PK11195 binding which is a marker of TPSO density and has been shown to be an indirect marker of inflammation in the same patients at the same timepoints and validated the BBB measurements against the blood‐CSF albumin ratio in a subset of the current study population.^13^ We included inclusion of two types of CSVD populations–sporadic CSVD and CADASIL–to study the range of presentations of CSVD. Lastly, the longitudinal design allowed us to investigate whether these two imaging measures predicted future cognitive impairment and atrophy risk.

This study also has limitations. Although ^11^C‐PK11195 is widely used as a marker of microglial signal, it may be confounded by off‐target and non‐specific tissue binding.^42^ It has been shown that ^11^C‐PK11195 binds to other inflammatory cells such as macrophages,^43^, ^44^ but the binding is weaker and more complex than with microglia.^45^ We accounted for the non‐specific binding where possible by controlling for endothelial binding in our analysis. Due to reliability issues with radiotracer production, not all patients were able to have PET scans. This, coupled with the relatively small size of the study in total and in each group, reduces the power of the study. TSPO binding has also been shown to relate to APOE ε4 status, which may have an effect on the results seen, unfortunately we do not have this information for the participants. Using MoCA as the measure of cognitive function is also limiting compared to a full cognitive battery with detailed tests for individual domains, but this test was chosen to minimize participant burden.

In conclusion, we found no evidence that increased BBB permeability predicts brain atrophy and cognitive impairment in CSVD. While we found no association between ^11^C‐PK11195 binding and future cognitive impairment, we did find an association with hippocampal atrophy, which could account for the well‐reported link between CSVD and the clinical exacerbation of neurodegenerative pathologies such as AD.

## CONFLICT OF INTEREST STATEMENT

The authors report no competing interests. Author disclosures are available in the supporting information.

## CONSENT STATEMENT

All participants provided written informed consent prior to participation in the study.

## Supporting information



Supporting Information

Supporting Information

## Data Availability

The data that support the findings of this study are available from the corresponding author, upon reasonable request.
