# Supplementary material for: Microglial reactivity predicts hippocampal, but not global, atrophy in cerebral small vessel disease
Source: Alzheimers Dement. 2026 Apr 2;22(4):e71336. doi: 10.1002/alz.71336 (PMC13052279; doi:10.1002/alz.71336)
Supplement: Supplementary file 1 — Supporting Information [file ALZ-22-e71336-s002.docx]

*Supplementary Table 1*. Comparison of baseline characteristics between participants excluded and included in brain atrophy linear regression analyses.

| **Variable** | **Neuroinflammatory markers (PET data)** | | | **Blood-brain barrier leakage markers (DCE-MRI data)** | | |
| --- | --- | --- | --- | --- | --- | --- |
|  | **Excluded from analysis**  **(N = 24)** | **Included in analysis**  **(N = 53)** | **p-value** | **Excluded from analysis**  **(N = 17)** | **Included in analysis**  **(N = 60)** | **p-value** |
| Female sex; n | 11 | 20 | 0.674 | 8 | 23 | 0.713 |
| Age at baseline, years | 63.81 | 65.95 | 0.556 | 70.16 | 63.90 | 0.080 |
| Education, years | 13.75 | 13.73 | 0.984 | 13.76 | 13.73 | 0.969 |
| WMH load (%) | 3.72 | 3.00 | 0.642 | 3.62 | 2.99 | 0.581 |
| Lacunes; n | 1.33 | 3.66 | 0.003 ** | 2.07 | 3.22 | 0.278 |
| Cerebral microbleeds; n | 0.62 | 6.19 | 0.010 * | 0.64 | 5.53 | 0.012 * |
| ^11^C-PK11195 global mean NAWM | -0.03 | -0.04 | 0.336 |  |  |  |
| ^11^C-PK11195 global mean all WM | -0.04 | -0.07 | 0.011 * |  |  |  |
| ^11^C-PK11195 hotspot volume NAWM | 10.9 | 15.3 | 0.381 |  |  |  |
| ^11^C-PK11195 hotspot volume all WM | 6.3 | 7.6 | 0.345 |  |  |  |
| BBB global mean NAWM |  |  |  | 1.7×10^−4^ | 4×10^−4^ | 0.064 |
| BBB global mean all WM |  |  |  | 1.7×10^−4^ | 3.8×10^−4^ | 0.090 |
| BBB hotspot volume NAWM |  |  |  | 3.35 | 5.48 | 0.369 |
| BBB hotspot volume all WM |  |  |  | 4.88 | 7.16 | 0.459 |

Values of continuous variables represent mean. NAWM = normal appearing white matter, WM = white matter, WMH = white matter hyperintensities. Continuous and categorical variables were compared using independent t-tests and chi-square tests respectively. * *p*<0.05, ** *p*<0.01

*Supplementary Table 2*. Comparison of baseline characteristics between participants excluded and included in Cox proportional hazards analyses with cognitive impairment.

| **Variable** | **Neuroinflammatory markers (PET data)** | | | **Blood-brain barrier leakage markers (DCE-MRI data)** | | |
| --- | --- | --- | --- | --- | --- | --- |
|  | **Excluded from analysis**  **(N = 32)** | **Included in analysis**  **(N = 45)** | **p-value** | **Excluded from analysis**  **(N = 27)** | **Included in analysis**  **(N = 50)** | **p-value** |
| Female sex; n | 12 | 19 | 1.000 | 10 | 21 | 1.000 |
| Age at baseline, years | 61.88 | 67.57 | 0.066 | 66.45 | 64.68 | 0.562 |
| Education, years | 13.90 | 13.63 | 0.693 | 13.34 | 13.94 | 0.378 |
| WMH load (%) | 2.42 | 3.22 | 0.178 | 2.05 | 3.31 | 0.018 * |
| Lacunes; n | 1.96 | 3.63 | 0.052 | 2.48 | 3.24 | 0.400 |
| Cerebral microbleeds; n | 1.32 | 6.61 | 0.032 * | 1.26 | 6.12 | 0.031 * |
| ^11^C-PK11195 global mean NAWM | -0.05 | -0.04 | 0.552 |  |  |  |
| ^11^C-PK11195 global mean all WM | -0.06 | -0.07 | 0.202 |  |  |  |
| ^11^C-PK11195 hotspot volume NAWM | 9.6 | 16.3 | 0.116 |  |  |  |
| ^11^C-PK11195 hotspot volume all WM | 6.0 | 7.9 | 0.225 |  |  |  |
| BBB global mean NAWM |  |  |  | 7.1×10^−4^ | 3.1×10^−4^ | 0.342 |
| BBB global mean all WM |  |  |  | 7.2×10^−4^ | 2.8×10^−4^ | 0.323 |
| BBB hotspot volume NAWM |  |  |  | 6.9 | 4.9 | 0.301 |
| BBB hotspot volume all WM |  |  |  | 8.5 | 6.6 | 0.442 |

Values of continuous variables represent mean. NAWM = normal appearing white matter, WM = white matter, WMH = white matter hyperintensities. Continuous and categorical variables were compared using independent t-tests and chi-square tests respectively. * *p*<0.05

*Supplementary Table 3*. Associations between conventional MRI markers and percentage **whole brain** volume change.

| Predictor | Whole group | | | Sporadic SVD | | | CADASIL | | |
| --- | --- | --- | --- | --- | --- | --- | --- | --- | --- |
|  | **β** | **95% CI** | **p-value** | **β** | **95% CI** | **p-value** | **β** | **95% CI** | **p-value** |
| Total brain volume | -0.05 | -0.30, 0.19 | 0.664 | -0.09 | -0.38, 0.20 | 0.537 | 0.33 | -0.17, 0.83 | 0.179 |
| Intracranial volume | 0.05 | -0.23, 0.33 | 0.724 | 0.02 | -0.31, 0.35 | 0.892 | 0.07 | -0.50, 0.64 | 0.790 |
| Gray matter volume | 0.09 | -0.17, 0.35 | 0.499 | -0.07 | -0.37, 0.24 | 0.652 | 0.43 | -0.06, 0.92 | 0.082 |
| All WM volume | 0.00 | -0.26, 0.27 | 0.977 | -0.07 | -0.36, 0.22 | 0.635 | -0.16 | -0.72, 0.39 | 0.533 |
| NAWM volume | 0.07 | -0.20, 0.34 | 0.616 | 0.08 | -0.23, 0.38 | 0.602 | -0.12 | -0.85, 0.62 | 0.741 |
| WMH | -0.03 | -0.28, 0.21 | 0.786 | -0.13 | -0.42, 0.16 | 0.373 | -0.05 | -0.70, 0.60 | 0.872 |
| WMH lesion load | -0.04 | -0.28, 0.20 | 0.745 | -0.10 | -0.40, 0.20 | 0.488 | -0.22 | -0.89, 0.44 | 0.486 |
| Number of lacunes | -0.30 | -0.53, -0.06 | 0.016 * | -0.28 | -0.56, 0.01 | 0.055 | -0.44 | -0.94, 0.06 | 0.080 |
| Number of CMBs | -0.03 | -0.28, 0.21 | 0.787 | -0.08 | -0.37, 0.21 | 0.563 | 0.36 | -0.17, 0.88 | 0.165 |

Table shows standardised β coefficients, 95% confidence intervals (CI) and p-values of linear regression models. Models have been adjusted for age and sex. NAWM = normal appearing white matter, WM = white matter, WMH = white matter hyperintensities, CMBs = cerebral microbleeds. * *p*<0.05

*Supplementary Table 4*. Associations between conventional MRI markers and percentage **hippocampal** volume change.

| Predictor | Whole group | | | Sporadic SVD | | | CADASIL | | |
| --- | --- | --- | --- | --- | --- | --- | --- | --- | --- |
|  | **β** | **95% CI** | **p-value** | **β** | **95% CI** | **p-value** | **β** | **95% CI** | **p-value** |
| Total brain volume | 0.49 | 0.27, 0.71 | 3.82×10^−5 ***^ | 0.65 | 0.41, 0.89 | 1.56×10^−6 ***^ | 0.16 | -0.43, 0.76 | 0.560 |
| Intracranial volume | -0.14 | -0.42, 0.15 | 0.345 | -0.17 | -0.51, 0.18 | 0.335 | -0.02 | -0.66, 0.62 | 0.952 |
| Gray matter volume | 0.32 | 0.06, 0.58 | 0.015* | 0.31 | 0.01, 0.62 | 0.043* | 0.35 | -0.23, 0.93 | 0.220 |
| All WM volume | -0.24 | -0.50, 0.03 | 0.076 | -0.23 | -0.53, 0.07 | 0.134 | -0.18 | -0.80, 0.44 | 0.552 |
| NAWM volume | -0.21 | -0.49, 0.06 | 0.122 | -0.25 | -0.56, 0.07 | 0.118 | -0.04 | -0.87, 0.79 | 0.913 |
| WMH | -0.01 | -0.26, 0.24 | 0.955 | 0.12 | -0.19, 0.43 | 0.435 | -0.26 | -0.97, 0.46 | 0.451 |
| WMH lesion load | -0.09 | -0.34, 0.16 | 0.480 | 0.00 | -0.32, 0.31 | 0.990 | -0.39 | -1.12, 0.33 | 0.268 |
| Number of lacunes | 0.02 | -0.24, 0.27 | 0.893 | 0.16 | -0.14, 0.46 | 0.292 | -0.17 | -0.79, 0.45 | 0.567 |
| Number of CMBs | 0.19 | -0.06, 0.44 | 0.133 | 0.06 | -0.24, 0.37 | 0.669 | 0.30 | -0.31, 0.90 | 0.310 |

Table shows standardised β coefficients, 95% confidence intervals (CI) and p-values of linear regression models. Models have been adjusted for age and sex. NAWM = normal appearing white matter, WM = white matter, WMH = white matter hyperintensities, CMBs = cerebral microbleeds. * *p*<0.05, ** *p*<0.01, *** *p*<0.001.

***Supplementary Table 5*. Associations between ^11^C-PK11195 binding and DCE-MRI markers with percentage WMH volume change over one year.**

| Predictor | Brain tissue | Whole group | | |
| --- | --- | --- | --- | --- |
|  |  | **β** | **95% CI** | **p-value** |
| ^11^PK11195 binding |  |  |  |  |
| Global mean | NAWM | -0.02 | -0.41, 0.37 | 0.921 |
|  | ALL WM | 0.03 | -0.38, 0.43 | 0.901 |
| Hotspot volume | NAWM | 0.32 | -0.14, 0.78 | 0.168 |
|  | ALL WM | -0.32 | -0.78, 0.13 | 0.155 |
| BBB permeability |  |  |  |  |
| Global mean | NAWM | 0.3 | -0.06, 0.66 | 0.099 |
|  | ALL WM | 0.3 | -0.06, 0.66 | 0.097 |
| Hotspot volume | NAWM | 0.3 | -0.1, 0.7 | 0.137 |
|  | ALL WM | 0.27 | -0.14, 0.67 | 0.187 |

Table shows standardised β coefficients, 95% confidence intervals (CI) and p-values of linear regression models. Models have been adjusted for age and sex. This analysis only included participants with Sporadic CSVD due to limited availability of WMH volume data at the longitudinal follow-up.

NAWM = normal appearing white matter, WM = white matter.

***Supplementary Table 6*. Associations between ^11^C-PK11195 binding and DCE-MRI markers with percentage mean diffusivity change over one year.**

| Predictor | Brain tissue | Whole group | | | Sporadic CSVD | | | CADASIL | | |
| --- | --- | --- | --- | --- | --- | --- | --- | --- | --- | --- |
|  |  | **β** | **95% CI** | **p-value** | **β** | **95% CI** | **p-value** | **β** | **95% CI** | **p-value** |
| ^11^PK11195 binding |  |  |  |  |  |  |  |  |  |  |
| Global mean | NAWM | 0 | -0.27, 0.28 | 0.975 | 0.11 | -0.22, 0.43 | 0.507 | -0.4 | -0.99, 0.19 | 0.16 |
|  | ALL WM | 0.06 | -0.22, 0.35 | 0.655 | 0.06 | -0.28, 0.39 | 0.735 | 0.74 | -0.11, 1.59 | 0.081 |
| Hotspot volume | NAWM | -0.22 | -0.49, 0.05 | 0.108 | -0.18 | -0.5, 0.14 | 0.258 | -0.5 | -1.07, 0.07 | 0.077 |
|  | ALL WM | -0.12 | -0.39, 0.15 | 0.365 | -0.07 | -0.4, 0.25 | 0.647 | -0.45 | -1.05, 0.15 | 0.127 |
| BBB permeability |  |  |  |  |  |  |  |  |  |  |
| Global mean | NAWM | -0.18 | -0.44, 0.09 | 0.191 | -0.27 | -0.58, 0.04 | 0.083 | 0.1 | -0.47, 0.66 | 0.719 |
|  | ALL WM | -0.18 | -0.44, 0.09 | 0.195 | -0.28 | -0.59, 0.03 | 0.078 | 0.13 | -0.43, 0.69 | 0.633 |
| Hotspot volume | NAWM | -0.2 | -0.48, 0.07 | 0.142 | -0.3 | -0.61, 0.01 | 0.055 | 0.03 | -0.6, 0.66 | 0.916 |
|  | ALL WM | -0.17 | -0.44, 0.11 | 0.232 | -0.26 | -0.57, 0.05 | 0.102 | 0.03 | -0.59, 0.65 | 0.919 |

Table shows standardised β coefficients, 95% confidence intervals (CI) and p-values of linear regression models. Models have been adjusted for age and sex. NAWM = normal appearing white matter, WM = white matter.

***Supplementary Table 7.* Associations between ^11^C-PK11195 binding and DCE-MRI markers with percentage fractional anisotropy change over one year.**

| Predictor | Brain tissue | Whole group | | | Sporadic CSVD | | | CADASIL | | |
| --- | --- | --- | --- | --- | --- | --- | --- | --- | --- | --- |
|  |  | **β** | **95% CI** | **p-value** | **β** | **95% CI** | **p-value** | **β** | **95% CI** | **p-value** |
| ^11^PK11195 binding |  |  |  |  |  |  |  |  |  |  |
| Global mean | NAWM | -0.05 | -0.33, 0.23 | 0.738 | -0.03 | -0.36, 0.3 | 0.845 | -0.21 | -0.87, 0.45 | 0.495 |
|  | ALL WM | 0.25 | -0.03, 0.53 | 0.082 | 0.27 | -0.06, 0.6 | 0.106 | 0.51 | -0.47, 1.48 | 0.271 |
| Hotspot volume | NAWM | 0.14 | -0.14, 0.42 | 0.316 | 0.22 | -0.1, 0.54 | 0.165 | -0.19 | -0.88, 0.5 | 0.543 |
|  | ALL WM | -0.03 | -0.3, 0.25 | 0.85 | 0 | -0.33, 0.33 | 0.996 | -0.23 | -0.92, 0.46 | 0.465 |
| BBB permeability |  |  |  |  |  |  |  |  |  |  |
| Global mean | NAWM | 0.3 | 0.04, 0.56 | 0.022 * | 0.37 | 0.07, 0.68 | 0.018 * | 0.02 | -0.52, 0.55 | 0.942 |
|  | ALL WM | 0.31 | 0.05, 0.56 | 0.022 * | 0.37 | 0.06, 0.68 | 0.02 * | 0.06 | -0.48, 0.6 | 0.81 |
| Hotspot volume | NAWM | 0.25 | -0.02, 0.52 | 0.064 | 0.33 | 0.02, 0.64 | 0.039 | -0.05 | -0.65, 0.55 | 0.859 |
|  | ALL WM | 0.22 | -0.05, 0.49 | 0.109 | 0.29 | -0.02, 0.61 | 0.067 | -0.05 | -0.64, 0.54 | 0.854 |

Table shows standardised β coefficients, 95% confidence intervals (CI) and p-values of linear regression models. Models have been adjusted for age and sex. NAWM = normal appearing white matter, WM = white matter.

Significant associations did not survive FDR correction (FDR-corrected *p* = 0.088)

***Supplementary Table 8*. Associations between ^11^C-PK11195 binding and DCE-MRI markers with percentage whole brain volume change.**

| Predictor | Brain tissue | Whole group | | | Sporadic CSVD | | | CADASIL | | |
| --- | --- | --- | --- | --- | --- | --- | --- | --- | --- | --- |
|  |  | **β** | **95% CI** | **p-value** | **β** | **95% CI** | **p-value** | **β** | **95% CI** | **p-value** |
| ^11^PK11195 binding |  |  |  |  |  |  |  |  |  |  |
| Global mean | NAWM | -0.28 | -0.6, 0.03 | 0.073 | -0.21 | -0.6, 0.17 | 0.267 | -0.41 | -1.03, 0.21 | 0.166 |
|  | ALL WM | -0.04 | -0.38, 0.3 | 0.818 | -0.1 | -0.5, 0.31 | 0.622 | 0.51 | -0.48, 1.5 | 0.275 |
| Hotspot volume | NAWM | -0.09 | -0.42, 0.24 | 0.584 | 0.01 | -0.4, 0.42 | 0.962 | -0.37 | -1.03, 0.29 | 0.239 |
|  | ALL WM | -0.18 | -0.5, 0.14 | 0.269 | -0.08 | -0.48, 0.32 | 0.685 | -0.4 | -1.06, 0.25 | 0.195 |
| BBB permeability |  |  |  |  |  |  |  |  |  |  |
| Global mean | NAWM | 0.15 | -0.15, 0.45 | 0.324 | 0.12 | -0.25, 0.5 | 0.505 | 0.16 | -0.42, 0.73 | 0.571 |
|  | ALL WM | 0.16 | -0.14, 0.46 | 0.294 | 0.13 | -0.25, 0.5 | 0.495 | 0.19 | -0.38, 0.77 | 0.481 |
| Hotspot volume | NAWM | 0.01 | -0.3, 0.31 | 0.958 | 0.03 | -0.36, 0.42 | 0.873 | -0.13 | -0.75, 0.48 | 0.644 |
|  | ALL WM | -0.01 | -0.31, 0.29 | 0.931 | 0 | -0.38, 0.39 | 0.981 | -0.13 | -0.73, 0.47 | 0.647 |

Table shows standardised β coefficients, 95% confidence intervals (CI) and p-values of linear regression models. Models have been adjusted for age and sex. NAWM = normal appearing white matter, WM = white matter.

***Supplementary Table 9*. Associations between ^11^C-PK11195 binding and DCE-MRI markers with percentage hippocampal volume change.**

| Predictor | Brain tissue | Whole group | | | Sporadic CSVD | | | CADASIL | | |
| --- | --- | --- | --- | --- | --- | --- | --- | --- | --- | --- |
|  |  | **β** | **95% CI** | **p-value** | **β** | **95% CI** | **p-value** | **β** | **95% CI** | **p-value** |
| ^11^PK11195 binding |  |  |  |  |  |  |  |  |  |  |
| Global mean | NAWM | -0.17 | -0.5, 0.17 | 0.317 | -0.09 | -0.48, 0.31 | 0.656 | -0.28 | -0.97, 0.41 | 0.381 |
|  | ALL WM | 0.1 | -0.24, 0.45 | 0.548 | -0.02 | -0.43, 0.38 | 0.905 | -0.07 | -1.18, 1.05 | 0.898 |
| Hotspot volume | NAWM | -0.35 | -0.67, -0.03 | 0.032 | -0.53 | -0.86, -0.2 | 0.003 | -0.02 | -0.77, 0.74 | 0.957 |
|  | ALL WM | -0.16 | -0.49, 0.18 | 0.341 | -0.13 | -0.53, 0.27 | 0.518 | -0.09 | -0.84, 0.67 | 0.798 |
| BBB permeability |  |  |  |  |  |  |  |  |  |  |
| Global mean | NAWM | 0.08 | -0.25, 0.41 | 0.643 | 0.03 | -0.36, 0.42 | 0.869 | 0.23 | -0.35, 0.81 | 0.413 |
|  | ALL WM | 0.11 | -0.22, 0.44 | 0.518 | 0.06 | -0.33, 0.45 | 0.772 | 0.26 | -0.32, 0.84 | 0.346 |
| Hotspot volume | NAWM | -0.13 | -0.46, 0.2 | 0.431 | -0.06 | -0.45, 0.34 | 0.77 | -0.1 | -0.73, 0.53 | 0.735 |
|  | ALL WM | -0.1 | -0.43, 0.22 | 0.525 | -0.01 | -0.4, 0.38 | 0.957 | -0.1 | -0.72, 0.52 | 0.724 |

Table shows standardised β coefficients, 95% confidence intervals (CI) and p-values of linear regression models. Models have been adjusted for age and sex. NAWM = normal appearing white matter, WM = white matter. * *p*<0.05, ** *p*<0.01, *** *p*<0.001.

Significant association did not survive FDR correction (adjusted p-value = 0.256).

***Supplementary Table 10*. Associations between ^11^C-PK11195 binding and DCE-MRI markers with cognitive impairment.**

| Predictor | Brain tissue | Whole group | | | Sporadic CSVD | | | CADASIL | | |
| --- | --- | --- | --- | --- | --- | --- | --- | --- | --- | --- |
|  |  | **Incident cases, n** | **Hazard ratio**  **[95% CI]** | **p-value** | **Incident cases, n** | **Hazard ratio**  **[95% CI]** | **p-value** | **Incident cases, n** | **Hazard ratio**  **[95% CI]** | **p-value** |
| ^11^PK11195 binding |  |  |  |  |  |  |  |  |  |  |
| Global mean | NAWM | 18/33 | 1.28 [0.79, 2.05] | 0.313 | 13/20 | 1.23 [0.74, 2.03] | 0.426 | 5/13 | 2.21 [0.7, 6.96] | 0.175 |
|  | ALL WM | 18/33 | 1.01 [0.61, 1.69] | 0.965 | 13/20 | 1.2 [0.63, 2.29] | 0.586 | 5/13 | 1.19 [0.38, 3.72] | 0.76 |
| Hotspot volume | NAWM | 18/33 | 1.07 [0.75, 1.54] | 0.709 | 13/20 | 0.94 [0.6, 1.47] | 0.79 | 5/13 | 3.96 [1.23, 12.69] | 0.021 * |
|  | ALL WM | 18/33 | 1.1 [0.76, 1.59] | 0.597 | 13/20 | 0.93 [0.61, 1.4] | 0.724 | 5/13 | 4.26 [1.23, 14.69] | 0.022 * |
| BBB permeability |  |  |  |  |  |  |  |  |  |  |
| Global mean | NAWM | 18/39 | 0.75 [0.29, 1.94] | 0.556 | 12/22 | 1.02 [0.33, 3.19] | 0.974 | 6/17 | 0 [0, 7.76] | 0.152 |
|  | ALL WM | 18/39 | 0.77 [0.25, 2.34] | 0.643 | 12/22 | 1.13 [0.29, 4.34] | 0.858 | 6/17 | 0 [0, 7.57] | 0.146 |
| Hotspot volume | NAWM | 18/39 | 0.74 [0.41, 1.32] | 0.309 | 12/22 | 0.94 [0.52, 1.71] | 0.846 | 6/17 | 0.01 [0, 3.31] | 0.118 |
|  | ALL WM | 18/39 | 0.73 [0.42, 1.27] | 0.263 | 12/22 | 0.96 [0.52, 1.77] | 0.903 | 6/17 | 0.09 [0, 2.2] | 0.138 |

Table shows standardised incident cases, hazard ratio, 95% confidence intervals (CI) and p-values of Cox proportional hazard models. Models have been adjusted for age and sex. The total number of patients included in the analyses is detailed in Figure 1. NAWM = normal appearing white matter, WM = white matter.

***Supplementary Table 11*. Associations between conventional MRI markers and cognitive impairment.**

| Predictor | Whole group | | | Sporadic CSVD | | | CADASIL | | |
| --- | --- | --- | --- | --- | --- | --- | --- | --- | --- |
|  | **Incident cases, n** | **Hazard ratio**  **[95% CI]** | **p-value** | **Incident cases, n** | **Hazard ratio**  **[95% CI]** | **p-value** | **Incident cases, n** | **Hazard ratio**  **[95% CI]** | **p-value** |
| Total brain volume | 25/54 | 0.79 [0.52, 1.21] | 0.276 | 18/36 | 0.85 [0.47, 1.56] | 0.609 | 7/18 | 0.56 [0.28, 1.15] | 0.114 |
| Intracranial volume | 24/53 | 0.8 [0.53, 1.22] | 0.302 | 18/36 | 0.82 [0.51, 1.32] | 0.416 | 6/17 | 0.58 [0.22, 1.54] | 0.276 |
| Gray matter volume | 25/54 | 0.66 [0.47, 0.94] | 0.02 | 18/36 | 0.47 [0.22, 1] | 0.049 | 7/18 | 0.46 [0.19, 1.11] | 0.085 |
| All WM volume | 24/53 | 1.02 [0.76, 1.38] | 0.876 | 17/35 | 0.96 [0.71, 1.31] | 0.806 | 7/18 | 1.46 [0.57, 3.69] | 0.429 |
| NAWM volume | 24/53 | 0.95 [0.66, 1.38] | 0.802 | 17/35 | 0.92 [0.58, 1.46] | 0.736 | 7/18 | 0.48 [0.16, 1.5] | 0.208 |
| WMH | 25/54 | 1.07 [0.76, 1.5] | 0.713 | 18/36 | 1.01 [0.73, 1.41] | 0.95 | 7/18 | 2.83 [0.89, 8.99] | 0.077 |
| WMH lesion load | 25/54 | 1.18 [0.81, 1.7] | 0.387 | 18/36 | 1.05 [0.74, 1.51] | 0.776 | 7/18 | 8.81 [1.4, 55.28] | 0.02 |
| Number of lacunes | 25/54 | 1.31 [0.98, 1.74] | 0.066 | 18/36 | 1.84 [1.06, 3.17] | 0.029 | 7/18 | 2.16 [1.05, 4.47] | 0.038 |
| Number of CMBs | 25/54 | 1.31 [0.91, 1.88] | 0.152 | 18/36 | 1.51 [1, 2.26] | 0.049 | 7/18 | 1.31 [0.53, 3.24] | 0.559 |

Table shows standardised hazard ratios, 95% confidence intervals (CI) and p-values of cox proportional hazards models. Models have been adjusted for age and sex. NAWM = normal appearing white matter, WM = white matter, WMH = white matter hyperintensities, CMBs = cerebral microbleeds. * *p*<0.05
